# An Ultra-Wideband Circularly Polarized Optically Transparent Antenna Using ITO Film

**DOI:** 10.3390/mi17020182

**Published:** 2026-01-29

**Authors:** Kunlun Wang, Mingyang Liu, Guang Lu, Hao Zhang

**Affiliations:** 1 School of Space Science and Technology, Shandong University at Weihai, Weihai 264209, China; wkl@sdu.edu.cn (K.W.); 202538111@mail.sdu.edu.cn (M.L.); 2 Laboratory for Electromagnetic Detection (LEAD), Institute of Space Sciences, Shandong University, Weihai 264209, China; 202437630@mail.sdu.edu.cn

**Keywords:** optically transparent, ultra-wideband monopole antenna, circularly polarized, ITO

## Abstract

This paper presents a novel broadband circularly polarized optically transparent monopole antenna using indium tin oxide (ITO) and PMMA. The proposed design successfully integrates ultra-wideband circular polarization characteristics with exceptional optical transparency. The antenna, constructed with a three-layer configuration utilizing ITO films as both the radiating patch and ground plane, along with transparent PMMA serving as the substrate, features compact dimensions of 40 × 40 × 1 mm^3^. By leveraging a co-optimized design incorporating a slotted hexagonal-ring radiating patch, triangular perturbation ground plane, and stepped-impedance feeding structure, the antenna achieves a circularly polarized operating bandwidth of 2.8–6.6 GHz (fractional bandwidth of 77.9%), with an axial ratio < 3 dB and return loss < −15 dB. The experimental findings exhibit strong consistency with the simulations, illustrating a high level of visible-light transmittance and radiation patterns characterized by right-hand circular polarization in the positive *z*-axis direction (+z) and left-hand circular polarization in the negative *z*-axis direction (−z). This innovative antenna shows great potential for applications in smart windows, display integration, and 5G communication systems.

## 1. Introduction

The rapid advancement of next-generation wireless technologies, including mobile communications, Internet-of-Things, and smart city systems, has generated increasingly demanding requirements for enhanced antenna performance [1]. Among these advancements, ultra-wideband circularly polarized (CP) antennas have gained significant attention for their ability to cover broader frequency ranges while effectively mitigating polarization mismatch and multipath interference. These advantages make them particularly valuable for applications in satellite communications, radio frequency identification, and mobile terminals [2,3]. Concurrently, the emergence of optically transparent antennas presents a promising solution owing to their distinctive features in visual camouflage and seamless environmental integration. Their potential applications span smart windows, display-embedded systems, and military stealth technologies [4,5,6]. Research on circularly polarized optically transparent antennas remains limited [6,7,8,9,10,11,12], with existing designs often suffering from structural complexity and narrow bandwidth. Hence, there is a pressing need to develop novel antenna designs that simultaneously achieve both wideband circular polarization characteristics and high optical transparency.

Monopole antennas have remained a research hotspot in the realm of ultra-wideband (UWB) antenna design due to their broadband potential. Significant bandwidth enhancement can be achieved through various approaches, including radiation patch optimization [13,14], ground plane modification [15,16], and innovative feeding structures [17]. Furthermore, techniques such as fractal geometries [18,19], parasitic element loading [20,21], and array configurations [22,23] can be employed to manipulate the surface current distribution, thereby enabling the realization of UWB circularly polarized (CP) monopole antennas. Conventional CP antennas typically rely on metallic radiating elements, which, despite their excellent electrical performance, suffer from inherent opacity, severely limiting their applicability in transparent scenarios. Additionally, achieving circular polarization typically requires intricate structural perturbations to induce the necessary phase difference, further complicating transparent antenna design. Therefore, a synergistic co-design approach that integrates material selection, structural ingenuity, and parametric optimization becomes indispensable for the concurrent attainment of wideband CP radiation and heightened optical transparency.

Indium tin oxide (ITO) is a type of transparent conductive oxide (TCO) material that exhibits high visible-light transmittance and moderate electrical conductivity [24,25]. Owing to its exceptional optical transparency, excellent chemical stability, and electromagnetic compatibility at microwave frequencies, ITO has emerged as a key functional material that effectively balances the dual requirements of optical transparency and electrical conductivity [26,27,28]. These unique properties make ITO the material of choice for transparent antenna design. However, research on ITO-based transparent circularly polarized (CP) antennas remains limited, with prevailing designs exhibiting narrow axial-ratio (AR) bandwidths. Existing transparent circularly polarized (CP) antennas face significant limitations: while ITO-based designs often adopt complex structures, they typically suffer from narrow axial ratio (AR) bandwidths; alternative conductive materials struggle to balance optical transmittance with electrical performance; and wideband versions usually rely on intricate hybrid structures, which increase fabrication complexity and compromise transparency. In this study, we address these challenges by applying the design methodology commonly used for conventional ultra-wideband (UWB) CP monopole antennas, but substituting the metal conductors with ITO films. The proposed antenna achieves an ultra-wide 3 dB AR bandwidth, high visible-light transmittance, and compact footprint—all through a simplified, co-optimized structure. This work effectively bridges the gap in practical transparent CP antenna technology, offering a versatile solution for diverse applications.

The remainder of this paper is organized as follows: Section 2 presents the antenna design methodology and theoretical analysis. Section 3 details the fabrication process and experimental characterization of the proposed antenna. Finally, comprehensive conclusions are drawn in Section 4.

## 2. Antenna Design and Analysis

**Figure 1:**
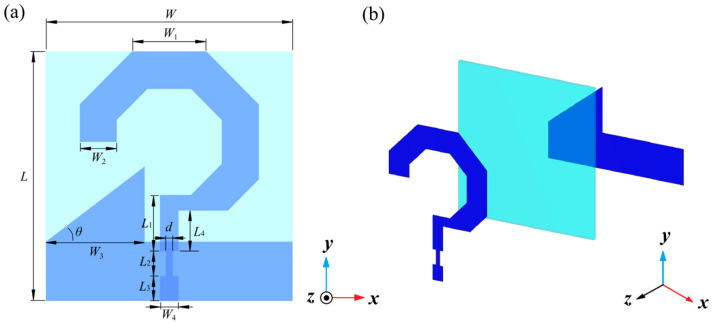
Structure of antenna: (**a**) Top view and (**b**) Layered view.

Figure 1 shows the structural schematic of the designed broadband circularly polarized optically transparent monopole antenna. The antenna features a three-layer configuration with overall dimensions of 40 mm × 40 mm × 1 mm. The main body consists of three layers: the top layer serves as the radiating patch, the bottom layer functions as the ground plane, and the middle layer acts as the dielectric substrate. Both the radiating patch and ground plane are fabricated using indium tin oxide (ITO) transparent conductive films, each with a sheet resistance of 3 Ω/sq. These films are positioned on the upper and lower surfaces of the dielectric substrate, respectively. The dielectric substrate employs a 1 mm-thick polymethyl methacrylate (PMMA) board with a relative permittivity of ε = 2.25. To enable broadband circular polarization, the antenna design incorporates specific features. The radiating element is structured as a notched regular hexagonal ring, while the ground plane integrates triangular structures in addition to the standard rectangular patch. The feeding network utilizes a stepped impedance transformer structure to effectively enhance the impedance matching bandwidth. Through parametric optimization of key antenna dimensions using CST Microwave Studio 2017 electromagnetic simulation software, the optimal structural parameters presented in Table 1 were ultimately determined. The time domain solver was adopted for the simulation, with open boundary conditions applied to mimic the free-space environment. The antenna model was meshed with hexahedral elements, and the mesh density was set to 20 cells per wavelength.

**Figure 2:**
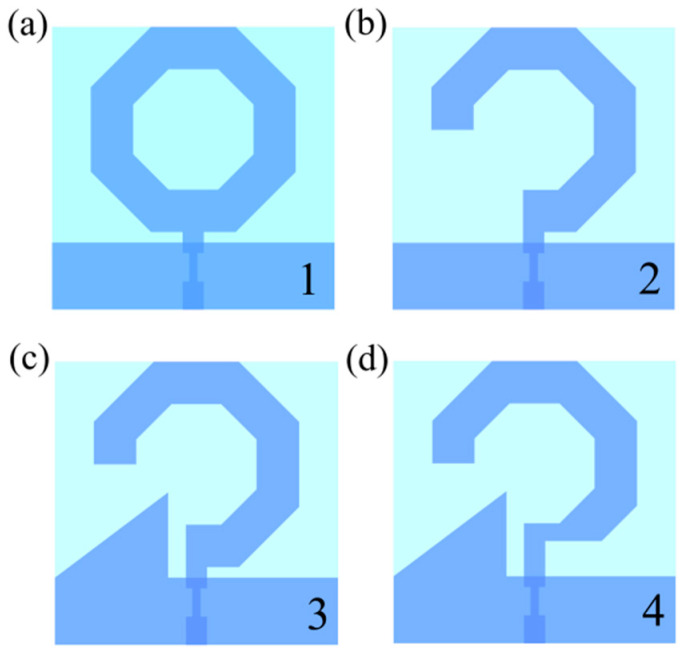
Design process of the proposed CP antenna. (**a**) Type 1. (**b**) Type 2. (**c**) Type 3. (**d**) Type 4.

Figure 2 illustrates the antenna design process. The baseline design (Antenna 1) consists of a regular hexagonal ring monopole antenna, as depicted in Figure 2a. Subsequently, the antenna design is refined by removing a quarter-section of the ring structure to form Antenna 2 (Figure 2b), resulting in changes to its radiation properties. To enhance circular polarization performance, Antenna 3 is developed by introducing a triangular perturbation structure on the ground plane (Figure 2c). This structural adjustment has a significant impact on the current distribution within the antenna, which is crucial for achieving circular polarization. Finally, Antenna 4 is realized by implementing corner truncations at the junction between the annular radiating patch and the microstrip feed line (Figure 2d). This optimization effectively improves the impedance bandwidth.

**Figure 3:**
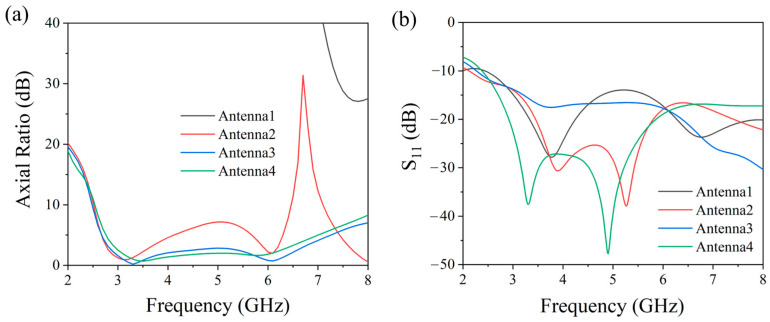
Simulated (**a**) AR and (**b**) $S_{11}$ curves of different antennas.

Figure 3 presents the comparative analysis of the axial ratio (AR) at the boresight (+z direction) and reflection coefficient ($S_{11}$) for different antenna configurations throughout the simulation-based design process. The analysis reveals that Antenna 1 exhibits AR values consistently above 3 dB across the 2–8 GHz frequency range, coupled with a narrow impedance bandwidth, indicating unsatisfactory circular polarization performance. Following structural refinements, Antenna 2 exhibits a notable enhancement in AR and an expansion of its operational bandwidth to higher frequencies. The introduction of a triangular perturbation structure in Antenna 3 yields superior circular polarization properties (AR < 3 dB) over a broad bandwidth, experimentally validating the critical role of perturbation in circular polarization enhancement, albeit with a minor compromise in reflection coefficient performance. Antenna 4 incorporates corner truncations at the radiating patch edges, effectively improving impedance matching while maintaining broadband circular polarization characteristics. The final optimized antenna achieves broadband circularly polarized operation across 2.8–6.4 GHz. This systematic optimization process clearly elucidates the impact of each structural modification on antenna performance characteristics.

**Figure 4:**
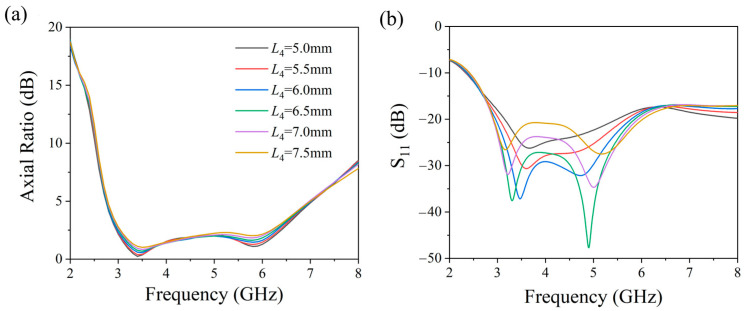
Effect of triangular structure variation on antennas (**a**) axial ratio and (**b**) reflection coefficient $S_{11}$.

**Figure 5:**
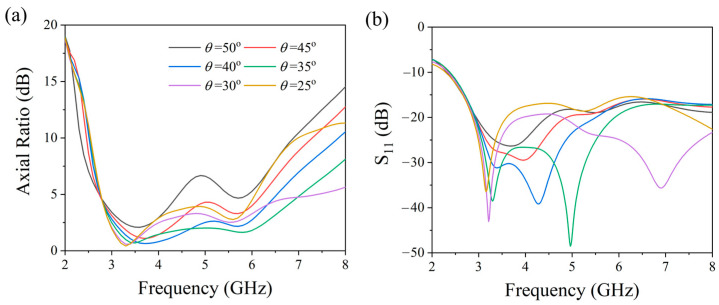
Effect of chamfer depth variation on antennas (**a**) axial ratio and (**b**) reflection coefficient $S_{11}$.

To explore how structural parameters affect antenna performance, we systematically analyzed the optimization process of the triangular angle *θ* and the truncation depth $L_{4}$ while maintaining other parameters constant. In our study, we examined how changes in *θ*, ranging from 50° to 25°, impacted antenna performance. Figure 4 visualizes the evolution of performance across the 2.8–6.4 GHz band as *θ* varied. We observed a continuous enhancement in the axial ratio (AR) as *θ* decreased from 50° to 35°, with optimal performance achieved at *θ* = 35°. However, reducing *θ* below 35° led to a degradation in AR characteristics, with a visible impact on the reflection coefficient. Figure 5 further examines the impact of truncation depth $L_{4}$ (5.0–7.5 mm). Interestingly, the AR demonstrated resilience to variations in $L_{4}$, exhibiting consistent circular polarization characteristics throughout the tested values. The reflection coefficient, on the other hand, showcased improvement with increasing $L_{4}$ within the 5.0–6.5 mm range, but began to deteriorate beyond 6.5 mm. Through co-optimization of AR bandwidth and impedance bandwidth, the optimal structural parameters listed in Table 1 were determined. This optimization process reveals distinct parameter–property relationships: the triangular angle *θ* primarily governs circular polarization characteristics, while the truncation depth $L_{4}$ mainly controls impedance matching performance.

.

**Figure 6:**
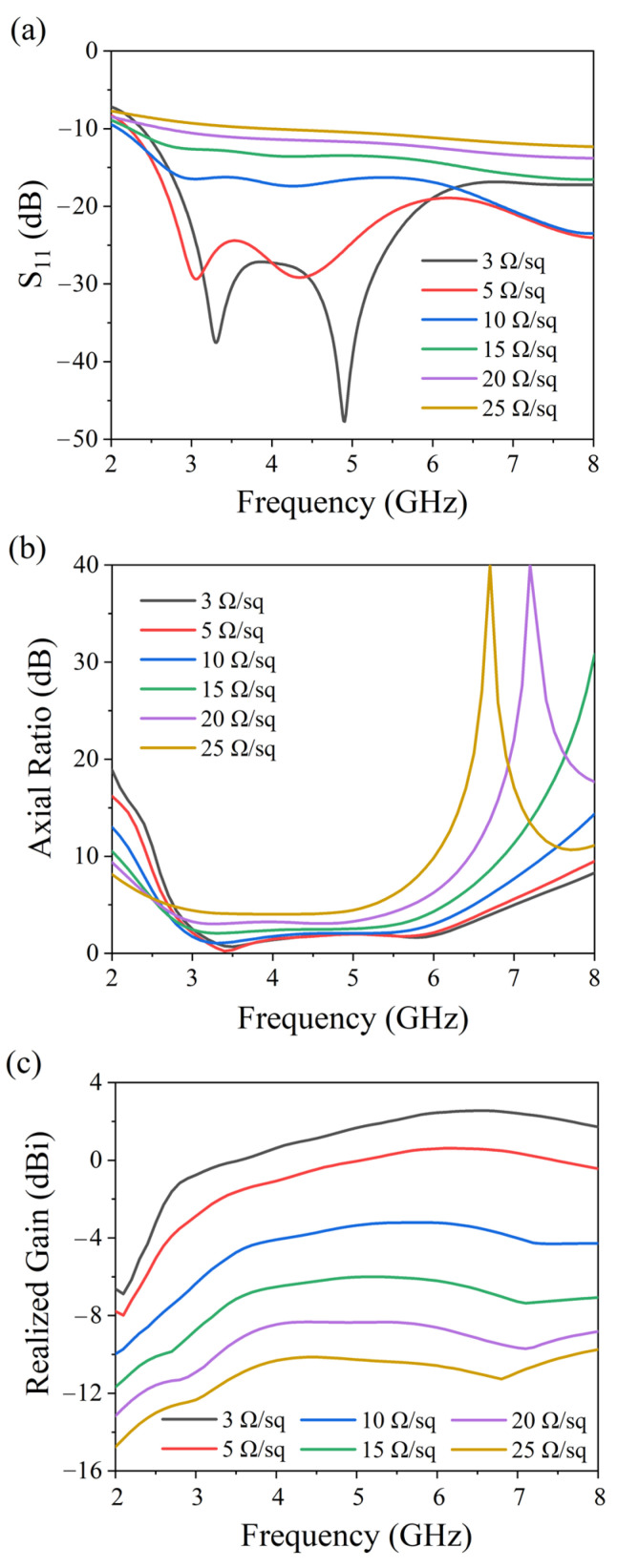
The influence of ITO material parameters on antennas’ (**a**) axial ratio, (**b**) reflection coefficient and (**c**) realized gain.

The influence of ITO material parameters on antenna performance was further analyzed. We simulated the performance of ITO-based antennas with different sheet resistance values ranging from 3 to 25 Ω/sq. As shown in Figure 6a, the $S_{11}$ parameter increases with higher sheet resistance. Figure 6b presents the simulated axial ratio, which also increases slightly with sheet resistance, though the overall effect remains relatively minor. In contrast, the antenna gain, shown in Figure 6c, decreases significantly as the sheet resistance rises. Therefore, to achieve better radiation performance, a lower sheet resistance of ITO is desirable. Considering practical fabrication constraints, an ITO thin film with a sheet resistance of 3 Ω/sq was selected.

**Figure 7:**
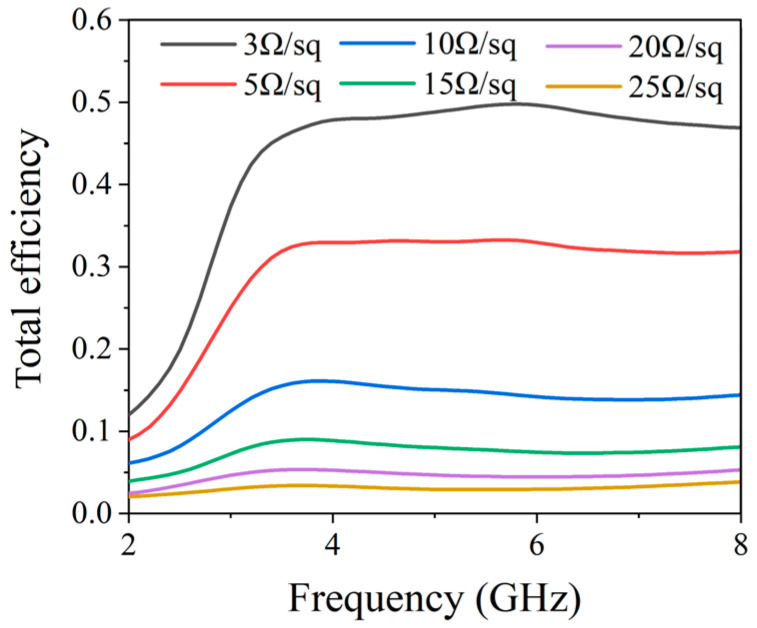
The influence of ITO material parameters on antennas’ total efficiency.

As shown in Figure 7, the simulated total radiation efficiency of the antenna under different ITO sheet resistances is presented. It is observed that as the ITO sheet resistance decreases, its ohmic loss gradually reduces, and the total radiation efficiency of the antenna shows a significant improvement trend. Notably, even when an ITO film with a low sheet resistance of 3 Ω/sq is adopted, the total radiation efficiency of the antenna remains relatively low. The core reasons include two aspects: first, as a transparent conductive oxide (TCO), ITO has an intrinsic electrical conductivity much lower than that of metallic conductors, inevitably leading to inherent ohmic loss which dissipates part of the input power as thermal energy; second, the influence of comprehensive losses in the antenna structure, including the slight dielectric loss of the PMMA substrate, the interface loss between the ITO film and the substrate, and the contact loss of the feeding structure. These factors collectively restrict the further improvement of efficiency. The aforementioned loss mechanisms directly result in the relatively low radiation gain of the antenna.

**Figure 8:**
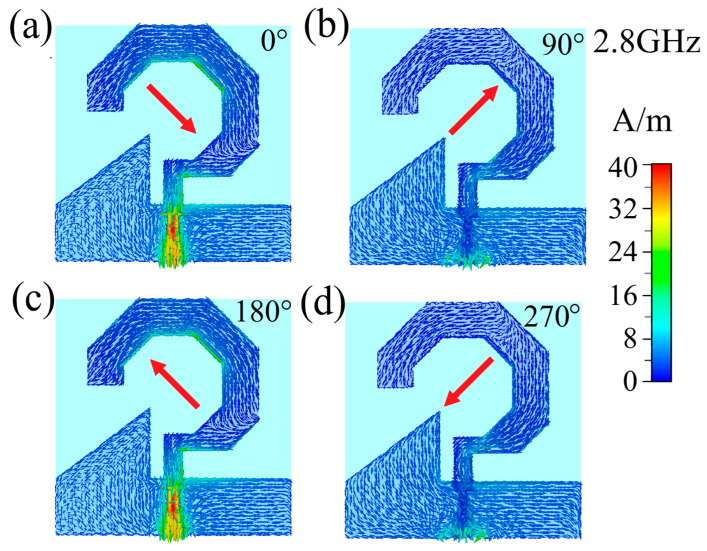
Surface current distribution of the 2.8 GHz antenna with feeding phases of: (**a**) 0°, (**b**) 90°, (**c**) 180°, and (**d**) 270°. The arrows show current flow direction; red arrows indicate the overall direction.

**Figure 9:**
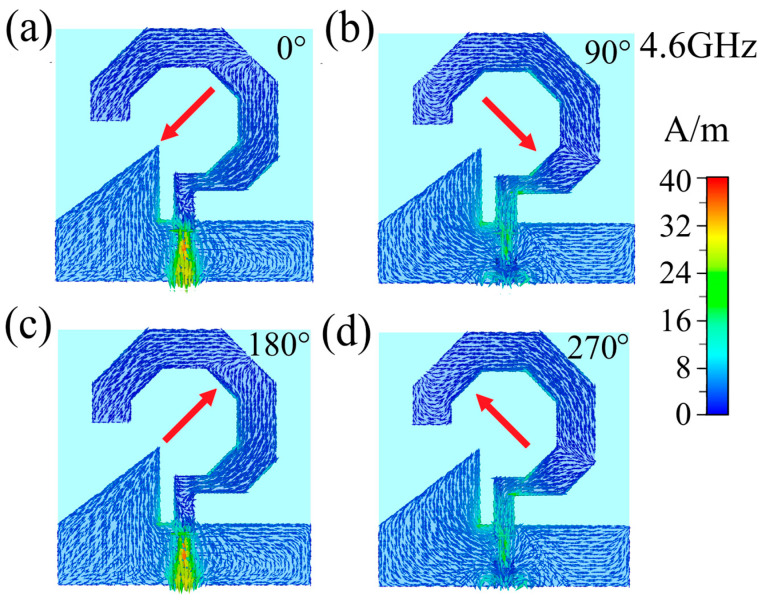
Surface current distribution of the 4.6 GHz antenna with feeding phases of: (**a**) 0°, (**b**) 90°, (**c**) 180°, and (**d**) 270°. The arrows show current flow direction; red arrows indicate the overall direction.

**Figure 10:**
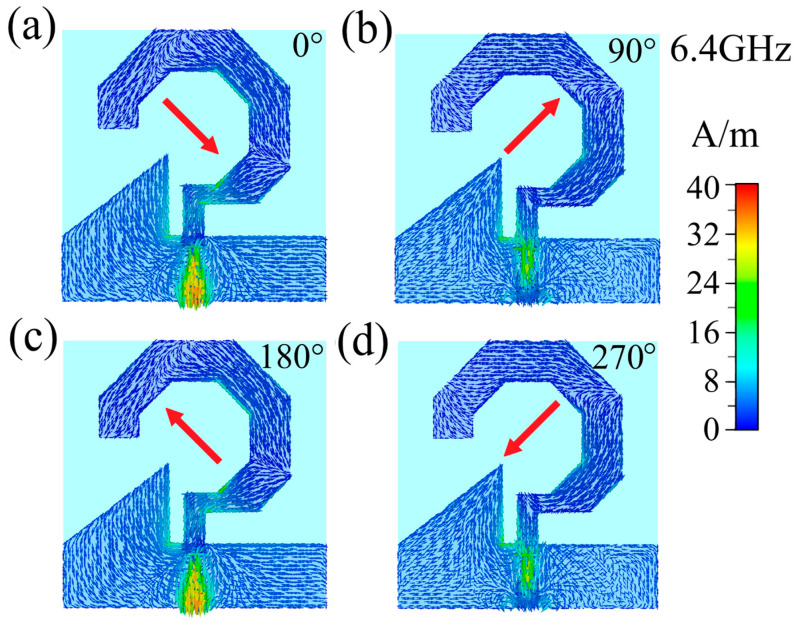
Surface current distribution of the 6.4 GHz antenna with feeding phases of: (**a**) 0°, (**b**) 90°, (**c**) 180°, and (**d**) 270°. The arrows show current flow direction; red arrows indicate the overall direction.

Circular polarization occurs when the electric field vector maintains a constant magnitude while rotating in a circular trajectory over time at any given observation point along the radiation direction. To elucidate the circular polarization mechanism of the proposed antenna, Figure 8, Figure 9 and Figure 10 present the simulated surface current distributions at 2.8 GHz, 4.6 and 6.4 GHz, respectively, showing four characteristic excitation phases (0°, 90°, 180°, and 270°) during one complete feed cycle, where the temporal evolution directly corresponds to progressive phase excitation. At 2.8 GHz (Figure 8), the resultant surface current vector exhibits distinct rotational behavior: oriented toward the upper-left quadrant at 0° phase, rotating to the lower-left at 90°, progressing to the lower-right at 180°, and completing the cycle at the upper-right quadrant at 270°. When observed from the +z-direction, this continuous counterclockwise rotation of surface currents leads to a corresponding counterclockwise rotation of the radiated electric field vector, thereby confirming the antenna’s operation in right-hand circular polarization (RHCP) mode at 2.8 GHz. Identical rotational characteristics are observed at 4.6 GHz (Figure 9) and 6.4 GHz (Figure 10), where the surface currents maintain the same counterclockwise rotational pattern, demonstrating consistent RHCP performance across both operational frequencies. These comprehensive analyses conclusively verify that the designed antenna functions as a right-hand circularly polarized radiator throughout its operating bandwidth.

**Figure 11:**
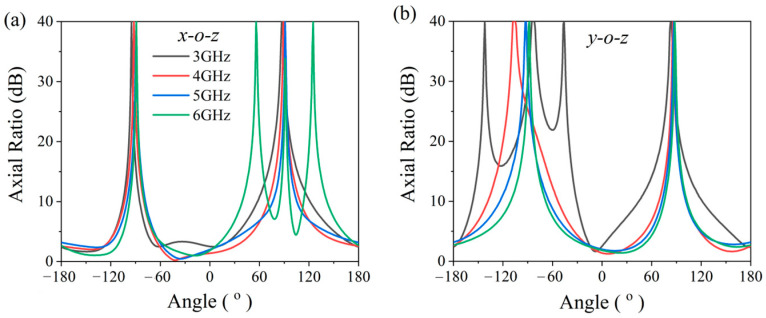
Simulated axial ratio radiation patterns of the antenna: (**a**) xoz plane and (**b**) yoz plane.

To further improve the validation of circular polarization (CP) performance, we have supplemented the analysis of AR characteristics. Figure 11 presents the simulated AR beamwidth patterns of the antenna at 3 GHz, 4 GHz, 5 GHz, and 6 GHz. The antenna exhibits excellent CP performance at the boresight direction. Within the 3 dB AR criterion, the AR beamwidths in the xoz plane are 34°, 98°, 77°, and 67° at 3 GHz, 4 GHz, 5 GHz, and 6 GHz, respectively, while those in the yoz plane are 18°, 58°, 66°, and 77° at the corresponding frequencies. These results demonstrate that the antenna possesses a wide angular coverage of CP characteristics in the main radiation direction.

## 3. Antenna Fabrication and Measurements

**Figure 12:**
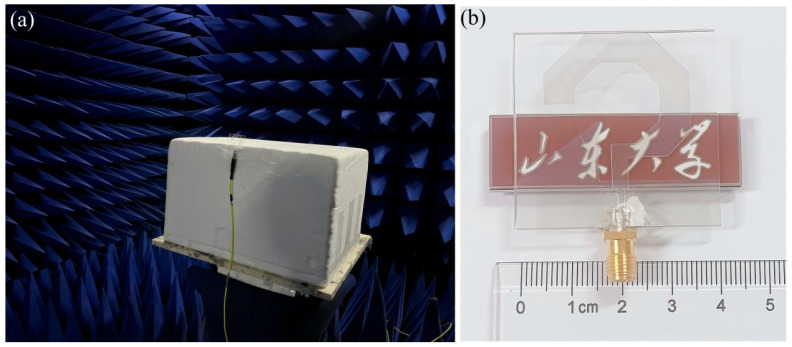
(**a**) Schematic diagram of antenna placement in anechoic chamber and (**b**) photograph of the antenna mounted on a university logo to demonstrate its transparency.

To validate the feasibility and reliability of the proposed design, a physical prototype was constructed in accordance with the optimized structural parameters. The antenna employs ITO transparent conductive films with a sheet resistance of 3 Ω/sq, precisely patterned into the designed hexagonal ring patch and triangular-modified ground plane. The ITO films (180 nm thick, sheet resistance 3 Ω/sq) were fabricated via magnetron sputtering on organic substrates, followed by annealing. This process ensures excellent surface flatness and stable electrical performance, critical for the antenna’s reliability. These conductive elements were then laminated on both sides of a 40 × 40 × 1 mm^3^ PMMA (polymethyl methacrylate) dielectric substrate. For signal transmission, a 50 Ω SMA coaxial connector was integrated, with the connector probe being firmly affixed to the microstrip feedline using a high-conductivity silver epoxy. The antenna performance characterization was conducted in a standard anechoic chamber environment. A Keysight PNA N5225B vector network analyzer (10 MHz–50 GHz) was employed to measure both S-parameters and radiation characteristics. Figure 12a demonstrates the antenna measurement setup in the anechoic chamber, while Figure 12b presents photographs of the fabricated prototype. The discernible visibility of background patterns through the antenna structure unequivocally attests to its superior optical transparency.

**Figure 13:**
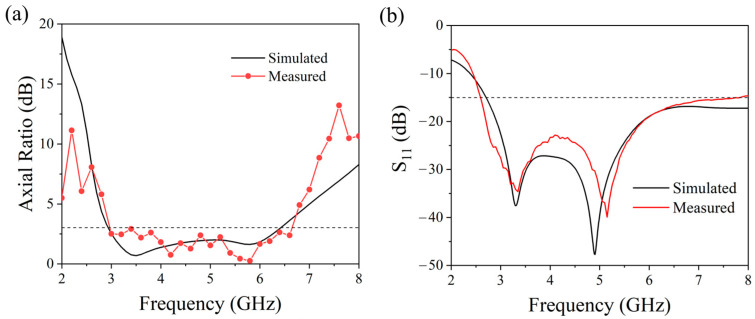
Comparison of measured and simulated results: (**a**) Antenna axial ratio and (**b**) S-parameters. The dashed lines represent the AR −3 dB and S11 −15 dB reference lines, respectively.

The linearly polarized horn antenna employed for axial ratio (AR) measurement is model LB-20180 from Ainfoinc, Chengdu, China with an operating frequency range of 2–18 GHz. Within the 2.8–6.6 GHz measurement band, the horn antenna exhibits stable gain and a cross-polarization greater than 30 dB, featuring excellent polarization purity. Prior to measurement, the Keysight PNA N5225B vector network analyzer from Santa Rosa, CA, USA was calibrated using the 85052D standard calibration kit to correct amplitude and phase errors in the measurement chain. A laser alignment tool was utilized to precisely align the transmitting horn antenna and the antenna under test along the same axis. The AUT was fixed on a precision rotation stage, while the horn antenna was mounted on an automatic polarization rotation platform, ensuring the rotation axis coincides with the signal propagation direction to avoid measurement errors induced by polarization misalignment. As shown in Figure 13a, the measured AR demonstrates excellent agreement with simulation results. The theoretical analysis predicts AR < 3 dB over 2.8–6.4 GHz (78.3% fractional bandwidth), while experimental results achieve AR < 3 dB across 2.9–6.6 GHz (77.9% fractional bandwidth). The S-parameters of the antenna, both simulated and measured, are presented in Figure 13b. Both sets of results indicate values that are below −15 dB within the axial ratio bandwidth frequency range. The measured data closely mirrors the simulated data, with minor discrepancies primarily ascribed to fabrication tolerances and measurement inaccuracies inherent in the antenna system.

**Figure 14:**
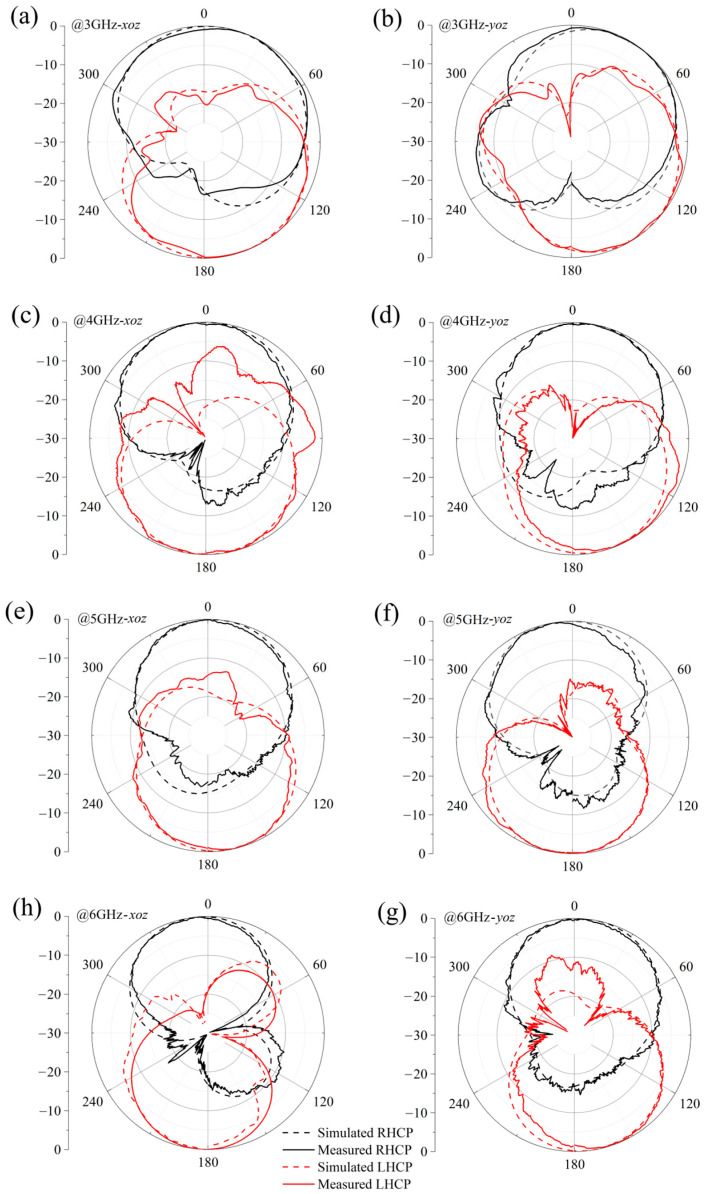
Measured vs. simulated normalized radiation patterns of the antenna: (**a**) 3 GHz-xoz plane, (**b**) 3 GHz-yoz plane, (**c**) 4 GHz-xoz plane, (**d**) 4 GHz-yoz plane, (**e**) 5 GHz-xoz plane, (**f**) 5 GHz-yoz plane, (**g**) 6 GHz-xoz plane, (**h**) 6 GHz-yoz plane.

In a controlled microwave anechoic chamber setting, a dual circularly polarized antenna operating within the 1–10 GHz frequency range was utilized as the emission source for assessing the circular polarization radiation patterns of the targeted antenna. Figure 14 presents normalized radiation pattern measurements at four characteristic frequencies (3 GHz, 4 GHz, 5 GHz, and 6 GHz), with radiation characteristics evaluated in both xoz and yoz principal planes. The measurements reveal distinct discrepancies between the Right-Hand Circularly Polarized (RHCP) and Left-Hand Circularly Polarized (LHCP) patterns, demonstrating clear bidirectional radiation features. Specifically, the antenna predominantly radiates RHCP waves in the +z-direction hemispace, while LHCP waves dominate in the −z-direction hemispace. The experimental patterns exhibit excellent agreement with theoretical predictions, validating the accuracy of the antenna design.

**Figure 15:**
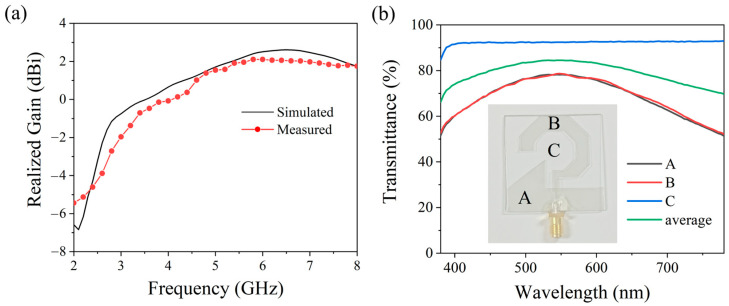
(**a**) Measured vs. Simulated Variation of Peak Gain with Frequency, (**b**) Measured visible light transmission spectrum of the antenna.

Figure 15a illustrates the frequency-dependent peak gain characteristic of the antenna. Experimental results show that within the 2.8–6.6 GHz operating band, the antenna gain increases monotonically from −2.7 dBi to 2.1 dBi as frequency rises. While the measured trend aligns well with the simulation data, the actual gain values are slightly lower than predicted, primarily due to factors such as fabrication tolerances and dielectric losses. Figure 15b presents the visible light transmission spectrum measured using a PerkinElmer LAMBDA 1050 spectrophotometer from Waltham, MA, USA. Three characteristic positions were tested: Points A and B with ITO coatings, and Point C without ITO. The measurements show that Point C maintains ~90% transmittance, while Points A and B exhibit > 50% transmittance, reaching up to 80% at peak. The green line in the figure represents the Area-weighted average transparency. These results underscore the remarkable optical transparency of the antenna structure in the visible light spectrum. The strong correlation between experimental results and theoretical predictions serves to validate the efficacy of the antenna design. As summarized in Table 2, the proposed antenna exhibits the widest 3 dB axial ratio bandwidth among the compared optically transparent circularly polarized designs, along with satisfactory optical transparency and a relatively simple structure.

Although the proposed ultra-wideband circularly polarized optically transparent antenna synergizes wide bandwidth and high optical transparency, it has limitations: Intrinsic ohmic loss of ITO films results in a lower peak gain (2.1 dBi) than traditional metallic antennas. An inherent trade-off exists between transparency and radiation efficiency—lower ITO sheet resistance (better conductivity/higher efficiency) requires thicker films, reducing transmittance, while thinner films for higher transparency increase resistance and ohmic loss. Additionally, the antenna’s 80% area-weighted average transparency meets most scenarios but can be improved for high-demand applications. Future optimizations include: (1) Adopting ITO composites with low-loss transparent conductive materials (e.g., silver nanowires, graphene) to reduce loss while maintaining high transparency; (2) Designing array structures to compensate for single-unit gain insufficiency via multi-unit superposition; (3) Optimizing ITO fabrication processes (e.g., sputtering/annealing parameters) to better balance conductivity and transparency.

## 4. Conclusions

This study introduces a novel broadband circularly polarized optically transparent antenna. The antenna features a simple overall design with compact dimensions of 40 mm × 40 mm × 1 mm. By employing ITO transparent conductive material for both the radiating patch and the ground plane, in conjunction with a transparent PMMA substrate, the antenna exhibits optical transparency in the visible light spectrum. Simulation results demonstrate high consistency with experimental measurements, showcasing a circular polarization operational bandwidth ranging from 2.8 GHz to 6.6 GHz (with a fractional bandwidth of 77.9%). This antenna effectively merges optical transparency with wideband circular polarization properties, holding promising application potentials in emerging communication devices, smart homes, smart cities, and various other fields.

## Figures and Tables

**Table 1:** Dimensions of the Antenna (Unit: mm).

*L*	*L*_1_	*L*_2_	*L*_3_	*L*_4_	*W*	*W*_1_	*W*_2_	*W*_3_	*W*_4_	*d*	*θ*
40.0	9.0	4.0	4.0	6.5	40.0	12.0	6.0	16	3	1.2	35°

**Table 2:** Comparison of the proposed antenna with existing antennas in the literature.

Ref.	Antenna Type	Conductive Layer	Size (mm)	Polaized	3 dB AR FBW	Peak Gain (dBi)	Transparency (%)	Mea. or Sim.
[5]	monopole	ITO	50 × 50	LP	5–7 GHz (33.3%)	−4 dBi	no	Measured
[8]	monopole	ITO	30 × 30	CP	3.34–3.44 GHz	0.6 dBi	83%	Measured
					4.07–4.56 GHz	1.3 dBi		
[9]	monopole	FTO	100 × 100	CP	860–970 MHz (12%)	1.4	78%	Measured
[10]	monopole	ITO	18.56 × 23.77	CP	7.9–13 GHz (48.9%)	3.85 dBi	no	Simulated
This work	monopole	ITO	40 × 40	CP	2.8–6.6 GHz (77.9%)	2.1	80	Measured

## Data Availability

The data that support the plots within this paper and other findings of this study are available from the corresponding authors on reasonable request.
